# Supplementary material for: Substance use, psychiatric symptoms, personal mastery, and social support among COVID-19 long haulers: A compensatory model
Source: PLoS One. 2023 Aug 3;18(8):e0289413. doi: 10.1371/journal.pone.0289413 (PMC10399860; doi:10.1371/journal.pone.0289413)
Supplement: Attachment — Submitted filename: responses to reviewer comments letter.docx [file pone.0289413.s001.docx]

We appreciate the opportunity to make revisions on our manuscript titled “Substance use, psychiatric symptoms, personal mastery, and social support among COVID-19 long haulers: A compensatory model” for consideration as an Research Article in *Plos One*. This manuscript represents original work, has not been under review elsewhere. The research was approved by the Institutional Review Boards of University of South Carolina. The authors do not have any conflicts of interest to report. All authors contributed to the manuscript at a level consistent with authorship and have approved the final version of the manuscript.

The editor and reviewer provided many insightful suggestions that we believe have improved the manuscript. Below are our detailed responses to reviewer’s comments (*in italics*).

Editor:

*When submitting your revision, we need you to address these additional requirements.*

*1. Please ensure that your manuscript meets PLOS ONE's style requirements, including those for file naming. The PLOS ONE style templates can be found at*

[*https://journals.plos.org/plosone/s/file?id=wjVg/PLOSOne_formatting_sample_main_body.pdf*](https://journals.plos.org/plosone/s/file?id=wjVg/PLOSOne_formatting_sample_main_body.pdf) *and*

[*https://journals.plos.org/plosone/s/file?id=ba62/PLOSOne_formatting_sample_title_authors_affiliations.pdf*](https://nam02.safelinks.protection.outlook.com/?url=https%3A%2F%2Fjournals.plos.org%2Fplosone%2Fs%2Ffile%3Fid%3Dba62%2FPLOSOne_formatting_sample_title_authors_affiliations.pdf&data=05%7C01%7Cctam%40mailbox.sc.edu%7Ca72a70fbdbc74337ab9e08db1f754b39%7C4b2a4b19d135420e8bb2b1cd238998cc%7C0%7C0%7C638138361215620928%7CUnknown%7CTWFpbGZsb3d8eyJWIjoiMC4wLjAwMDAiLCJQIjoiV2luMzIiLCJBTiI6Ik1haWwiLCJXVCI6Mn0%3D%7C3000%7C%7C%7C&sdata=Kg0%2BRIdrDyWAN2SxUCGzBUGByay6tl1Xn2dA7Se0H6g%3D&reserved=0)

Responses: Thanks for the instructions. We have proofread the manuscript in line with PLOS ONE’s style requirements.

*2. In your Methods section, please include additional information about your dataset and ensure that you have included a statement specifying whether the collection and analysis method complied with the terms and conditions for the source of the data.*

Responses: Thanks for the suggestions. Data in the current study were collected by us and the collection and analysis process complied with the study protocol approved by the University of South Carolina Institutional Review Board, including obtaining online written consent prior to the participation and deidentification of the data. We have now noted in the manuscript on page 5.

*3. Please provide additional details regarding participant consent. In the ethics statement in the Methods and online submission information, please ensure that you have specified what type you obtained (for instance, written or verbal, and if verbal, how it was documented and witnessed). If your study included minors, state whether you obtained consent from parents or guardians. If the need for consent was waived by the ethics committee, please include this information.*

*Once you have amended this/these statement(s) in the Methods section of the manuscript, please add the same text to the “Ethics Statement” field of the submission form (via “Edit Submission”).*

Responses: Thanks for catching that. We have now noted that online written consent was obtained from the participants prior to the surveys (on page 5). No minors were included in the study given the inclusion criteria of 18 years of age or older. As requested, we have added the text to the submission form.

For additional information about PLOS ONE ethical requirements for human subjects research, please refer to [http://journals.plos.org/plosone/s/submission-guidelines#loc-human-subjects-research](https://nam02.safelinks.protection.outlook.com/?url=http%3A%2F%2Fjournals.plos.org%2Fplosone%2Fs%2Fsubmission-guidelines%23loc-human-subjects-research&data=05%7C01%7Cctam%40mailbox.sc.edu%7Ca72a70fbdbc74337ab9e08db1f754b39%7C4b2a4b19d135420e8bb2b1cd238998cc%7C0%7C0%7C638138361215620928%7CUnknown%7CTWFpbGZsb3d8eyJWIjoiMC4wLjAwMDAiLCJQIjoiV2luMzIiLCJBTiI6Ik1haWwiLCJXVCI6Mn0%3D%7C3000%7C%7C%7C&sdata=MGPmLjRzdNJxoQ3B38jVF0y%2BdkKNGxKDO8rYfDtlmEY%3D&reserved=0).

*4. Thank you for stating in your Funding Statement:*

*“The research was supported in part by the by the National Institute of Allergy and Infectious Diseases of the National Institutes of Health under award number R01AI127203-5S1. The content is solely the responsibility of the authors and does not necessarily represent the official views of the National Institutes of Health.”*

*Please provide an amended statement that declares *all* the funding or sources of support (whether external or internal to your organization) received during this study, as detailed online in our guide for authors at* [*http://journals.plos.org/plosone/s/submit-now*](http://journals.plos.org/plosone/s/submit-now)*.  Please also include the statement “There was no additional external funding received for this study.” in your updated Funding Statement.*

*Please include your amended Funding Statement within your cover letter. We will change the online submission form on your behalf.*

Responses: Given no additional funding support for this study, we have clarified that in the statement as requested (see below). The amended statement is also provided in the cover letter.

‘The research was supported in part by the by the National Institute of Allergy and Infectious Diseases of the National Institutes of Health under award number R01AI127203-5S1. The content is solely the responsibility of the authors and does not necessarily represent the official views of the National Institutes of Health. There was no additional external funding received for this study.’

*5. We note that you have indicated that data from this study are available upon request. PLOS only allows data to be available upon request if there are legal or ethical restrictions on sharing data publicly. For more information on unacceptable data access restrictions, please see* [*http://journals.plos.org/plosone/s/data-availability#loc-unacceptable-data-access-restrictions*](http://journals.plos.org/plosone/s/data-availability#loc-unacceptable-data-access-restrictions)*.*

*In your revised cover letter, please address the following prompts:*

*a) If there are ethical or legal restrictions on sharing a de-identified data set, please explain them in detail (e.g., data contain potentially sensitive information, data are owned by a third-party organization, etc.) and who has imposed them (e.g., an ethics committee). Please also provide contact information for a data access committee, ethics committee, or other institutional body to which data requests may be sent.*

*b) If there are no restrictions, please upload the minimal anonymized data set necessary to replicate your study findings as either Supporting Information files or to a stable, public repository and provide us with the relevant URLs, DOIs, or accession numbers. For a list of acceptable repositories, please see* [*http://journals.plos.org/plosone/s/data-availability#loc-recommended-repositories*](http://journals.plos.org/plosone/s/data-availability#loc-recommended-repositories)*.*

*We will update your Data Availability statement on your behalf to reflect the information you provide.*

Responses: Thanks for the instructions. As requested, a dataset including variables of the current study has now been available on a repository. Below is the link to the data:

<https://figshare.com/articles/dataset/SPSS_dataset_of_substance_use_in_COVID_long_haulers_sav/22285330>

*6. Please review your reference list to ensure that it is complete and correct. If you have cited papers that have been retracted, please include the rationale for doing so in the manuscript text, or remove these references and replace them with relevant current references. Any changes to the reference list should be mentioned in the rebuttal letter that accompanies your revised manuscript. If you need to cite a retracted article, indicate the article’s retracted status in the References list and also include a citation and full reference for the retraction notice.*

Responses: As requested, we have reviewed the in-text citations and reference thoroughly in the manuscript.
 *Thank you very much for your submission to PLOS ONE, short titled "Substance use and psychiatric and psychosocial factors among COVID-19 long haulers". I am sorry for the delay in the review - it was difficult to locate reviewers. Your manuscript has been accepted with minor revisions. They are listed below.*

Responses: We appreciate the editors and reviewers’ positive feedback on our manuscript. Also thanks for the valuable comments and suggestions, which have improved the manuscript. Please see our detailed responses to reviewers’ comments.

REVIEWER 1

*• The paper is well-written and engaging. It is appropriately sourced and referenced.
• Authors have no bias.
• The study adequately addresses issues of potential gender bias.*

Responses: Thanks for the positive comments.

*• Strongly recommend that authors replace the term “substance misuse” with “substance use” throughout. The term “misuse” is now considered very pejorative (like the term “substance abuse”) and implies deliberate intentionality.*

Responses. Thanks for the suggestion. We have now used the term “substance use” throughout the manuscript.

• Study strength – analytic strategy and techniques used. Very well formulated and executed.

Responses. Thanks for noting this.

*• Study weakness – sampling technique – a sample of convenience using social media sources. This creates potential respondent bias compared to a randomly generated response sample.*

Responses. We agree with the reviewer that the current study would be limited by the sampling approach (convenient sampling on social media). We have discussed the limitations and suggested that future research should use a randomized sampling approach and integrated forms of data (e.g., self-reported and electronic health records) to investigate substance use and its protective and risk factors in COVID-19 long haulers (on page 12).

REVIEWER 2

*• Overall, the paper is about an interesting topic and the analysis is well described.
• In my opinion the paper deserves to be published in a better journal than Plos One. (for this the analysis should be extended and the presentation of the model improved)*

Responses: Thanks for the comments.

*• In general, write all numbers as 0.38 and not as .038!*

Responses: We have made revisions as requested.

*• ...there have been 87,899,721 total cases of COVID-19 (4). Is this unique cases? Please clarify.*

Responses: Thanks for the question. This is the number of cumulative COVID-19 infection cases worldwide as of July 6, 2022. We have now clarified it in the manuscript on page 2.

*• The figures of the SEMs are very helpful and informative. If the figures could be in color that would be great!*

Responses: Thanks for the suggestion. We have colored the figures for better presentations.

*• Results section 3.4 is too short and more explanations should be given about the analysis.*

Responses: Thanks for the comment. In order to report concise and objective information in the results section, we left the explanation and interpretation of the SEM results in the discussion section. However, we briefly elaborated them a bit by noting the role of risk factors and protective factors based on the coefficients (on page 10).

*• It would be interesting to study the effect of the size of the data. To do this, subsampling could be used to study the effect on the results. What is the minimal sample size that allows to obtain the same results?*
Responses: Thanks for the suggestion. We have considered this suggested test, but we think it seems to be beyond the scope of our study and hesitate to do the analysis for the following consideration. Given the uncertainty of the representativeness and homoscedasticity across subsamples, the suggested analyses, by being run multiple times, would increase the threat to the Type I or Type II errors, affecting the robustness of the findings. Accordingly, the suggested analyses might not provide meaningful improvements to the study.

However, according to Kline (2010), the minimum sample size for SEM can be determined by the number of manifest factors, suggesting a minimum of 10 cases per one factor. In our structural model, we have a total of 18 manifest factors, implying a need of a minimum sample size of 180 to reach enough statistical power. The sample size in the current study was 460, which exceeded the requirement.

Ref:

Kline, R. (2010). *Principles and practice of structural equation modeling*. *Structural Equation Modeling*. https://doi.org/10.1038/156278a0

We appreciate the editor’s and reviewers’ insightful comments and believe these revisions have strengthened the manuscript.
